# Advances in hydrogen-rich water for plant abiotic stress management: antioxidant regulation, hormonal crosstalk, and signal integration

**DOI:** 10.3389/fpls.2026.1898691

**Published:** 2026-07-24

**Authors:** Lei Huang, Ying Hu, Yi Wang, Xiao-huan Xia, Xu-feng Ding, Peng-ke Zhao

**Affiliations:** Shaoxing Academy of Agricultural Sciences, Shaoxing, Zhejiang, China

**Keywords:** abiotic stress tolerance, antioxidant defense, hydrogen-rich water, molecular hydrogen, plant stress tolerance, reactive oxygen species, selective antioxidant, signal transduction

## Abstract

Abiotic stresses such as salinity, heavy metal toxicity, drought, and extreme temperatures severely limit plant growth and agricultural productivity by disrupting cellular homeostasis and inducing excessive reactive oxygen species (ROS) accumulation. Hydrogen-rich water (HRW), has emerged as a promising eco-friendly strategy for enhancing plant stress tolerance. This review synthesizes current knowledge on HRW-mediated stress alleviation, offering an integrated framework of antioxidant regulation, hormonal crosstalk, and signal transduction. HRW confers protection through selective scavenging of cytotoxic radicals while preserving signaling ROS, upregulates enzymatic and non-enzymatic antioxidants to maintain redox balance, regulates ion homeostasis and osmolyte accumulation, and protects chloroplast and mitochondrial integrity. Furthermore, HRW modulates gene expression and stress-responsive pathways via interactions with phytohormones and gaseous signaling networks. This integrated approach distinguishes the present work by bridging previously dispersed mechanistic insights across multiple stress types. Despite promising findings, challenges remain regarding hydrogen perception mechanisms, application standardization, and field-level validation. Advancing these areas will support the integration of HRW into sustainable agricultural practices for improved crop resilience.

## 1 Introduction

Abiotic stresses such as soil salinization, heavy metal toxicity, extreme temperatures, and drought severely disrupt plant physiological processes including photosynthesis, respiration, and nutrient uptake, leading to substantial crop yield losses (Gechev and Petrov, 2020). It is estimated that abiotic stresses account for more than 50% yield loss in major crops worldwide, posing a serious threat to global food security (Oshunsanya et al., 2019; Junaid and Gokce, 2024). Under abiotic stress conditions, the metabolic balance of reactive oxygen species (ROS) in plants is disrupted. Under normal physiological conditions, ROS function as signaling molecules involved in plant growth and development. However, under stress conditions, excessive ROS accumulation triggers membrane lipid peroxidation, protein denaturation, and DNA damage, ultimately leading to programmed cell death or even whole-plant mortality (Bhuyan et al., 2020; Maurya, 2020). Therefore, the development of efficient, safe, and environmentally friendly strategies for enhancing plant stress tolerance is of great practical significance for agricultural production.

Hydrogen (H_2_) was long considered a physiologically inert gas without biological functions. This notion was overturned in 2007 by the Japanese scholar Ohsawa and his colleagues, who discovered for the first time that hydrogen gas can selectively scavenge the most cytotoxic hydroxyl radical (·OH) within cells, while having no significant effect on reactive oxygen species that play roles in signal transduction, such as hydrogen peroxide (H_2_O_2_) (Ohsawa et al., 2007). This finding marked the beginning of a new era in the study of the biological effects of hydrogen and provided an important theoretical foundation for subsequent research on plant stress tolerance.

HRW is typically prepared by bubbling high-purity hydrogen gas (≥99.99%) into distilled or deionized water until saturation (approximately 1.6 mg H_2_ L^−^¹ at 20 °C) (Ohsawa et al., 2007). Alternatively, HRW can be generated through water electrolysis or by dissolving hydrogen-producing chemical tablets (e.g., metallic magnesium or magnesium hydride) in water (Zhao et al., 2021a). The dissolved H_2_ concentration is commonly quantified using a hydrogen sensor or gas chromatography, and the prepared solution should be used immediately or stored in airtight containers to minimize H_2_ escape (Hancock, 2022). It should be noted that H_2_ concentrations are reported in the original units used in the cited studies (e.g., mg/L, μM, or saturation percentages).

HRW has attracted considerable attention in plant stress research due to its unique selective antioxidant properties, which allow it to scavenge highly cytotoxic radicals without interfering with signaling ROS (Li et al., 2022; Hancock, 2022). Several features distinguish HRW from conventional stress-relief agents: its safety and eco-friendliness allow application without residue concerns; its low cost makes it accessible for large-scale agricultural use; and its gaseous nature enables rapid diffusion across cell membranes. Given the rapid expansion of this field and the growing demand for sustainable agricultural solutions, a systematic synthesis of the physiological and molecular mechanisms underlying HRW-mediated stress tolerance is both timely and warranted.

In plant science, subsequent studies revealed that endogenous H_2_ levels increase under stress conditions (Cui et al., 2013). Exogenous H_2_ is typically applied as hydrogen-rich water (HRW) due to its low solubility (Hancock, 2022). A growing body of evidence has demonstrated that HRW effectively alleviates various abiotic stresses across diverse plant species (Wang et al., 2024; Ye et al., 2025; Huang et al., 2026). However, a systematic synthesis of HRW-induced stress tolerance mechanisms—particularly from the perspectives of antioxidant defense and signal transduction—remains lacking.

This review aims to critically evaluate the role of HRW in mitigating major abiotic stresses in plants by integrating physiological, biochemical, and molecular evidence into a coherent framework of antioxidant regulation, hormonal crosstalk, and signal transduction, thereby connecting mechanistic insights that have previously been addressed separately across the literature. It focuses on elucidating antioxidants and signaling mechanisms, including ion regulation and phytohormone interactions. Additionally, it identifies current research limitations and highlights future directions to facilitate the development of efficient, standardized, and field-applicable hydrogen-based strategies for sustainable crop stress management.

## 2 Alleviating effects of HRW on major abiotic stresses

**Figure 1:**
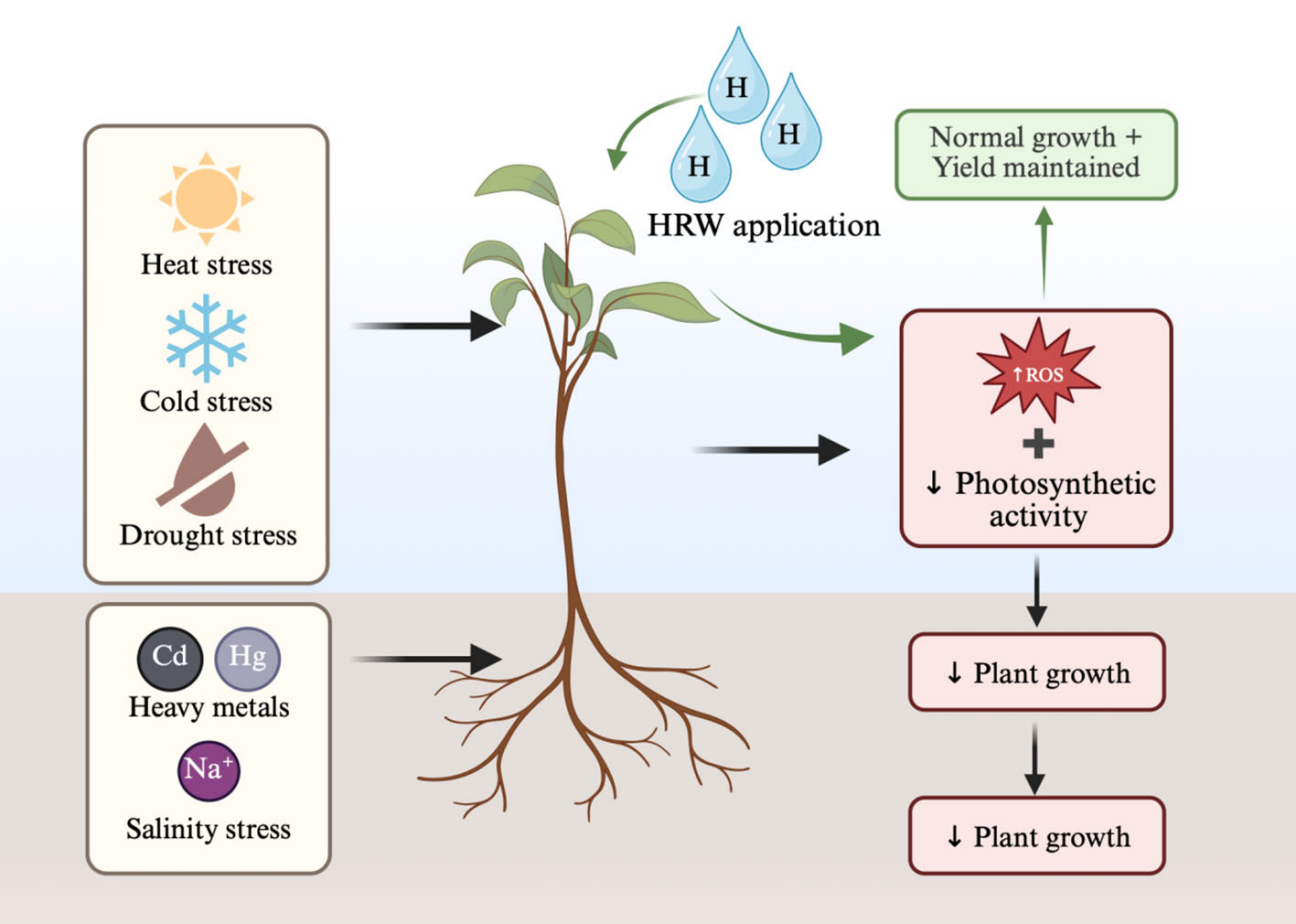
Schematic overview of HRW-mediated protection against abiotic stress-induced damage. Abiotic stresses (drought, salinity, heavy metals, and extreme temperatures) trigger excessive ROS accumulation in plants, leading to growth inhibition and yield loss. HRW treatment alleviates this damage by scavenging cytotoxic ROS and restoring normal growth.

HRW has been widely reported to alleviate a broad range of abiotic stresses in various plant species (Cui et al., 2014; Wu et al., 2020; Huang et al., 2026). As shown in **Figure 1**, HRW protects plants from abiotic stress-induced damage by scavenging cytotoxic ROS and restoring normal growth. The following sections provide a systematic overview of the physiological effects and molecular mechanisms by which HRW confers protection against salt stress, heavy metal toxicity, temperature stress, drought, ultraviolet B (UV-B) radiation, and postharvest preservation. A comprehensive summary of HRW responses, including representative plant species and key physiological effects under each stress condition, is presented in **Table 1**.

**Table 1:** Summary of HRW-mediated protection against major abiotic stresses in plants.

Stress type	Primary mechanism	Key physiological responses	Species	References
Salt stress	Ion homeostasis + Antioxidant	K^+^/Na^+^ homeostasis, antioxidant enzyme activity	*Oryza sativa*	(Wu et al., 2015)
Salt stress	Ion homeostasis + Antioxidant	K^+^/Na^+^ homeostasis, SOS1-mediated Na^+^ efflux	*Hordeum vulgare*	(Wu et al., 2020)
Salt stress	Antioxidant + Ion homeostasis	ROS scavenging, SOS pathway, ASA-GSH cycle	*Cucumis sativus*	(Yu et al., 2023)
Salt stress	Ion homeostasis + Antioxidant + Cell wall	Ion transport, antioxidant enzymes, cell wall biosynthesis	*Fragaria × ananassa*	(Wang et al., 2024)
Salt stress	Hormone signaling	Strigolactone biosynthesis, root growth	*Solanum lycopersicum*	(Ye et al., 2025)
Heavy metal stress (Cd)	Ion transport + Antioxidant	Cd uptake reduction, GSH metabolism	*Brassica chinensis*	(Wu et al., 2021a)
Heavy metal stress (Cd)	Antioxidant + Redox	GSH metabolism, redox homeostasis	*Medicago sativa*	(Cui et al., 2013)
Heavy metal stress (Hg)	Ion transport + Antioxidant	Hg accumulation reduction, ROS burst alleviation	*Medicago sativa*	(Cui et al., 2014)
Heavy metal stress (Al)	Antioxidant + Nutrient homeostasis	Redox homeostasis, nutrient homeostasis	*Zea mays*	(Zhao et al., 2017)
Low temperature stress	Antioxidant	Antioxidant enzyme activity, ASA-GSH cycle	*Cucumis sativus*	(Wang et al., 2023)
Low temperature stress	Secondary metabolism	Phenylpropanoid and flavonoid pathways	*Tetrastigma hemsleyanum*	(Liu et al., 2022)
High temperature stress	Antioxidant + Photosynthesis	Photosynthetic capacity, *HSP70* expression, osmoprotectants	*Cucumis sativus*	(Chen et al., 2017a)
Drought stress	Antioxidant + Transcription	Antioxidant enzymes, *Dreb1* upregulation, reduced cell death	*Triticum aestivum*	(Islam et al., 2023)
Drought stress	Antioxidant + Metabolism	Sugar metabolism, ASA-GSH cycle	*Hordeum vulgare*	(Song et al., 2023)
Drought stress	Hormone signaling	ABA biosynthesis and signaling, stomatal closure	*Solanum lycopersicum*	(Huang et al., 2026)
Drought stress	Hormone signaling + Ion homeostasis	Apoplastic pH regulation, stomatal sensitivity to ABA	*Medicago sativa*	(Wu et al., 2021b)
Ultraviolet radiation	Secondary metabolism	(Iso)flavonoid metabolism, antioxidant defense	*Medicago sativa*	(Xie et al., 2015)
Ultraviolet radiation	Secondary metabolism	Phenylpropanoid pathway, isoflavone enrichment	*Glycine max*	(Xu et al., 2024)
Ultraviolet radiation	Secondary metabolism + Calcium signaling	Anthocyanin accumulation, calcium signaling	*Raphanus sativus*	(Su et al., 2014)
Postharvest preservation	Hormone signaling + Antioxidant	Ethylene inhibition, cell wall integrity, antioxidant capacity	*Actinidia chinensis*	(Hu et al., 2014)
Postharvest preservation	Antioxidant	Antioxidant enzyme activity, ROS reduction	*Hypsizygus marmoreus*	(Chen et al., 2017b)
Postharvest preservation	Hormone signaling + Antioxidant	Melatonin and phytohormone regulation	*Abelmoschus esculentus*	(Dong et al., 2023b)
Postharvest preservation	Ion homeostasis + Metabolism	Ion balance, aquaporin regulation, sugar metabolism	*Lilium davidii*	(Liu et al., 2023)

### 2.1 Salt stress

Salt stress inhibits plant growth through three primary mechanisms: osmotic stress, ion toxicity (excessive accumulation of Na^+^/Cl^−^), and subsequent oxidative stress. Numerous studies have demonstrated that HRW is an effective approach for alleviating salt-induced damage in crops, with its mechanisms primarily involving the maintenance of ion homeostasis and activation of the antioxidant system (Wu et al., 2020; Yang et al., 2024; Yu et al., 2023).

Regarding ion homeostasis, HRW treatment significantly increases the K^+^/Na^+^ ratio in the roots of crop seedlings such as rice, alfalfa, and tomato. This is achieved by inhibiting excessive Na^+^ uptake while promoting K^+^ retention, thereby maintaining normal cellular osmotic pressure and physiological metabolism (Wu et al., 2015; Hu et al., 2014). Electrophysiological studies have revealed that HRW effectively maintains intracellular K^+^/Na^+^ homeostasis by promoting salt overly sensitive 1(*SOS1)*-mediated Na^+^ efflux from roots and preventing K^+^ loss induced by membrane depolarization (Wu et al., 2020). At the level of seed germination, HRW pretreatment significantly improves the germination rate and seedling survival of rice seeds under salt stress and increases the K^+^/Na^+^ ratio in both shoots and roots (Xu et al., 2013; Cui et al., 2014). Furthermore, HRW enhances plant salt tolerance by regulating the expression of ion transport-related genes and acting synergistically with signaling molecules such as melatonin (Su et al., 2021). Meanwhile, HRW induces the synthesis and accumulation of osmolytes such as proline and soluble sugars, enhancing cellular water retention capacity and alleviating osmotic damage (Wang et al., 2025; Musazade et al., 2025). In strawberry, HRW treatment not only increases leaf relative water content and reduces electrolyte leakage but also decreases Na^+^ translocation from roots to shoots (Wang et al., 2024).

In terms of antioxidant defense, excessive ROS induced by salt stress are the primary cause of oxidative cellular damage. In ‘Flame Seedless’ grapes (*Vitis vinifera L*.), HRW treatment significantly upregulates the activities of antioxidant enzymes such as superoxide dismutase (SOD), peroxidase (POD), and catalase (CAT), while enhancing fruit quality parameters including total soluble solids, soluble sugars, and anthocyanin content (Ali et al., 2025). In fragrant rice, HRW treatment (foliar spray or irrigation) enhances SOD, POD, and CAT activities and reduces malondialdehyde (MDA) content (Fu et al., 2020). HRW also activates α/β-amylase activity in rice seeds, accelerates soluble sugar accumulation, and upregulates the expression of antioxidant enzyme genes, thereby alleviating oxidative damage (Xu et al., 2013). In cucumber seedlings, HRW significantly enhances ROS scavenging capacity and maintains cellular redox balance by inhibiting the expression of the ROS-producing protein kinase Rho-like GTPase 1 (*ROP1*), upregulating SOS signaling pathway-related genes, and key genes of the ascorbate-glutathione (ASA-GSH) cycle, including glutathione reductase (*GR*) and ascorbate peroxidase 2 (*APX2*) (Yu et al., 2023).

In alfalfa and leguminous crops, the combined application of HRW and selenium exhibits synergistic stress resistance effects (Zhao et al., 2017); in cucumber and tomato, HRW regulates the expression of antioxidant enzyme genes and synergistically coordinates ion homeostasis and antioxidant systems to effectively alleviate salt damage (Lin et al., 2014; Huang et al., 2026). Notably, the combined application of HRW and the salt-tolerant plant growth-promoting rhizobacteria (PGPR) strain *Cytobacillus firmus* L71 synergistically enhances salt tolerance in *Pennisetum giganteum* by downregulating negative regulators such as *CaM*/*CML* and *JAZ* and upregulating positive regulators such as *A-ARR*, providing a novel strategy for saline-alkali soil remediation (Chu et al., 2025). Furthermore, emerging evidence indicates that HRW enhances salt tolerance by modulating phytohormone signaling; specifically, hydrogen gas promotes root growth in tomato seedlings under salt stress through upregulating strigolactone biosynthesis genes *SlD27* and *SlMAX1* (Ye et al., 2025). Collectively, HRW enhances salt tolerance by coordinating ion homeostasis, antioxidant defense, osmolyte accumulation, and signaling pathways, thereby improving plant growth under salinity stress conditions.

### 2.2 Heavy metal stress

Heavy metal contamination, including cadmium (Cd), mercury (Hg), lead (Pb), and aluminum (Al), is a major environmental issue in agricultural soils (Rashid et al., 2023). Heavy metals cause plant growth inhibition and physiological damage by inducing ROS bursts and interfering with the uptake and transport of essential mineral elements (Kumar et al., 2022). Molecular H_2_, owing to its multiple biological activities including antioxidant effects, stress resistance, and residue-free properties, has been recognized as a green approach for heavy metal detoxification (Alwazeer et al., 2025). As a donor of H_2_, HRW exerts its detoxifying effects against heavy metal stress primarily through three aspects: blocking heavy metal uptake, alleviating oxidative damage, and maintaining ion homeostasis.

HRW can effectively block the uptake of heavy metals such as Cd and Pb by plant roots through downregulating the expression of metal ion transporter genes, while also inhibiting their translocation from roots to shoots, thereby significantly reducing heavy metal accumulation in plant tissues (Ma et al., 2021). In Chinese cabbage, HRW treatment blocked the Cd-induced upregulation of *IRT1*and *Nramp1* (responsible for Cd uptake), enhanced the expression of *HMA3* (regulating Cd sequestration into root vacuoles), and suppressed the expression of *HMA2* and *HMA4* (responsible for Cd transport to xylem), resulting in significantly reduced Cd content in both roots and shoots (Wu et al., 2015). Similarly, in pak choi, HRW reduced Cd uptake by downregulating *BcIRT1* and *BcZIP2* expression and altering their ion uptake selectivity (Wu et al., 2021a). In fragrant rice, HRW treatment not only promoted seedling growth and increased CAT and POD activities but also significantly reduced Pb and Cd contents in roots (Ma et al., 2021).

Regarding mercury (Hg) toxicity, HRW treatment alleviated HgCl_2_-induced ROS bursts and growth inhibition in alfalfa seedlings while reducing Hg accumulation (Cui et al., 2014). In Cd-As co-contaminated soil, HRW treatment increased leaf biomass and Cd concentration in *Solanum nigrum*, improved the phytoremediation efficiency for Cd and As, and simultaneously reduced soil pH and Cd bioavailability (Chen et al., 2025). Furthermore, HRW prepared with ammonia borane (NH_3_·BH_3_) enhanced the tolerance of rapeseed seedlings to various stresses including CdCl_2_ and reduced Cd accumulation, a process dependent on nitric oxide (NO) signaling produced via the nitrate reductase (*NR*) pathway (Zhao et al., 2021a). In *Arabidopsis*, hydrogen gas can also construct a physical barrier by altering cell wall pectin content, further restricting Cd transcellular transport (Li et al., 2022).

Overall, HRW mitigates heavy metal toxicity by limiting metal uptake, promoting sequestration (i.e., vacuolar compartmentalization and cell wall binding of heavy metal ions), reprogramming transporter expression, and reducing oxidative injury in plant tissues.

### 2.3 Temperature stress

Extreme temperatures (low and high) disrupt cell membrane structure, interfere with metabolic enzyme activities, and inhibit photosynthesis. HRW exerts significant protective effects against both low- and high-temperature stress. Under low-temperature stress, plants counteract damage through osmotic adjustment, antioxidant activation, and cold-responsive gene expression (Li et al., 2022). In cucumber, a cold-sensitive crop, low-temperature stress severely inhibits growth and reduces photosynthetic efficiency (Zhang et al., 2025). HRW treatment alleviates chilling-induced damage by promoting growth and photosynthetic efficiency while reducing membrane lipid peroxidation and ROS accumulation. The protective mechanisms involve activation of antioxidant enzymes at both activity and transcriptional levels, enhancement of the AsA-GSH cycle, and maintenance of cellular redox homeostasis (Wang et al., 2023). Seed soaking with HRW enhances cold resistance by strengthening the antioxidant system and improving osmotic adjustment via proline and soluble sugar accumulation (Liu et al., 2017). More broadly, plant cold tolerance is achieved through the accumulation of osmoregulators, activation of antioxidant defenses, and rebalancing of phytohormone networks. At the molecular level, transcription factors such as C-repeat binding factor (*CBF*), myeloblastosis transcription factor (*MYB*), and WRKY-domain transcription factor (*WRKY*) activate cold-responsive genes to enhance cold tolerance (Zhang et al., 2025). In *Tetrastigma hemsleyanum*, HRW regulates cold tolerance by modulating phenylpropanoid and flavonoid biosynthesis pathways, suppressing the cold-induced upregulation of key biosynthetic genes including phenylalanine ammonia-lyase (*PAL*), chalcone synthase (*CHS*), caffeic acid O-methyltransferase (*COMT*), and cinnamoyl-CoA reductase (*CCR*), as well as the accumulation of their associated metabolites (Liu et al., 2022).

Under high-temperature stress, HRW pretreatment alleviates heat-induced damage in cucumber seedlings by improving photosynthetic capacity, increasing chlorophyll content and chlorophyll fluorescence parameters, reducing electrolyte leakage and lipid peroxidation, and maintaining membrane stability. Recent studies have highlighted that precise regulation of carbon partitioning and source-sink relations plays a critical role in maintaining crop productivity under heat stress (Zhao et al., 2025). These protective effects are associated with increased activities of antioxidative enzymes, promoted accumulation of osmoprotectants (e.g., proline, soluble sugars), and upregulation of heat shock protein heat shock protein 70 (*HSP70)* expression (Chen et al., 2017a), consistent with broader findings on carbon partitioning and metabolic adjustments in heat stress adaptation (Zhao et al., 2025). These findings are consistent with earlier reports that HRW pretreatment enhances thermotolerance by improving photosynthetic capacity, increasing antioxidant responses, and promoting HSP70 and osmolyte accumulation (Li et al., 2022). The protective effects of HRW under both low- and high-temperature stress are consistently linked to protection of the photosynthetic apparatus and activation of the antioxidant system, highlighting the universal nature of its stress-regulatory function.

Taken together, HRW confers resilience to temperature extremes by preserving photosynthetic efficiency, stabilizing membranes, inducing heat shock proteins, and sustaining metabolic activity under stress.

### 2.4 Drought stress

HRW treatment effectively alleviates drought-induced injury across various plant species. Studies consistently show that HRW promotes seed germination and seedling establishment under drought conditions. In wheat and barley, HRW treatment enhanced germination rate, vigor index, relative water content, and chlorophyll content, while activating antioxidant enzymes and osmoregulatory substance accumulation (Islam et al., 2023; Song et al., 2022). Mechanistic studies revealed that HRW elevates α-/β-amylase activities and re-establishes redox homeostasis through the ascorbate-glutathione cycle (Song et al., 2023).

The interplay between H_2_ and abscisic acid (ABA) signaling has been extensively investigated. In tomato, HRW enhanced drought tolerance by increasing photosynthetic capacity, antioxidant enzyme activities, and endogenous ABA content via upregulation of ABA biosynthesis genes including zeaxanthin epoxidase (*SlZEP*), 9-cis-epoxycarotenoid dioxygenase (*SlNCED*), and abscisic aldehyde oxidase (*SlAAO*) and signaling components (*SlSnRK2*, *SlAREB*) (Huang et al., 2026; Yan et al., 2022). HRW also reduced stomatal aperture and water loss, while optimizing carbon allocation and fruit quality (Huang et al., 2026). In alfalfa, HRW pretreatment enhanced stomatal sensitivity to ABA by increasing apoplastic pH in an H^+^-ATPase-dependent manner (Wu et al., 2021b). Additionally, HRW prepared with ammonia borane conferred drought tolerance in rapeseed seedlings via nitric oxide signaling (Zhao et al., 2021a). Collectively, these findings demonstrate that HRW enhances drought tolerance through a multi-pathway network involving ABA signaling, antioxidant defense, osmoregulation, and NO signaling. Recent advances in crop drought resistance have further highlighted the importance of ABA signaling and stomatal regulation in mediating plant adaptation to water deficit (He et al., 2024). Beyond protecting growing plants, HRW also preserves postharvest quality under low-temperature storage by reducing ROS accumulation, enhancing antioxidant enzyme activities, and maintaining cellular energy status (Dong et al., 2023a). Overall, HRW strengthens drought adaptation by modulating ABA-dependent stomatal behavior, improving water retention, enhancing carbon allocation, and maintaining cellular redox equilibrium.

### 2.5 Ultraviolet radiation

HRW treatment effectively alleviates oxidative damage induced by UV-B radiation. In alfalfa, HRW pretreatment enhances plant tolerance to UV-B by modulating the (iso)flavonoid metabolic profile, increasing the accumulation of antioxidant compounds, and activating the antioxidant defense system (Xie et al., 2015). In germinated soybean, H_2_ alleviates UV-B stress by regulating the activities and gene expression of key enzymes in the phenylpropanoid metabolic pathway, promoting the enrichment of isoflavones (particularly aglycones), while directly or indirectly scavenging reactive oxygen species (Xu et al., 2024).

Furthermore, HRW also exhibits significant regulatory effects under ultraviolet A (UV-A) radiation. In radish sprouts, HRW promotes anthocyanin accumulation by regulating ROS homeostasis, activating antioxidant enzymes, and upregulating the expression of anthocyanin biosynthesis-related genes (Su et al., 2014). Transcriptomic analysis further revealed that HRW regulates multiple pathways including light signal perception, secondary metabolite biosynthesis, and hormone signaling (Zhang et al., 2018a). Mechanistic studies indicate that the inositol trisphosphate (*IP_3_*)-dependent calcium signaling pathway is involved in this regulatory process (Zhang et al., 2018b). Under different light spectra, HRW combined with blue light or UV-A treatment further enhances the activities of *CHS* and UDP-glucose flavonoid 3-O-glucosyltransferase (*UFGT*) enzymes, promotes anthocyanin accumulation, and improves antioxidant capacity (Zhang et al., 2019). In soybean sprouts, HRW promotes ascorbic acid accumulation by upregulating the expression of genes involved in ascorbic acid biosynthesis and recycling (Jia et al., 2017).

### 2.6 Postharvest preservation

HRW has emerged as a green preservation strategy for postharvest horticultural products, extending shelf life and maintaining quality through multiple regulatory mechanisms (Zuo et al., 2025). HRW treatment inhibits ethylene biosynthesis, reduces respiration rate, and delays softening by regulating cell wall metabolism and energy homeostasis (Fang et al., 2024; Hu et al., 2014). In various horticultural products, HRW enhances antioxidant capacity by activating antioxidant enzymes (SOD, CAT, APX, GR) and increasing non-enzymatic antioxidant contents, while reducing ROS accumulation and membrane lipid peroxidation (Chen et al., 2017b; Dong et al., 2023a; Hu et al., 2014). In fresh-cut kiwifruit, HRW combined with slightly acidic electrolyzed water maintained firmness, color, and nutritional quality, and reduced microbial counts (Zhao et al., 2021b). In okra, HRW upregulated melatonin biosynthetic genes and increased melatonin content, while modulating phytohormones (increased IAA and GA, decreased ABA), thereby delaying senescence (Dong et al., 2023b). In chestnuts, HRW combined with carboxymethyl chitosan/sodium alginate coating enhanced hydrogen retention efficiency and provided both antioxidant and physical barrier protection (Dong et al., 2025). In lily scales, HRW maintained ion balance (increased K^+^/Na^+^ ratio), regulated aquaporin gene expression, and increased sugar-metabolizing enzyme activities, thereby enhancing water retention capacity and prolonging storage period (Liu et al., 2023). Collectively, these findings demonstrate that HRW preserves postharvest quality through a multi-pathway network involving antioxidant defense, energy metabolism, cell wall integrity, ethylene inhibition, phytohormone regulation, and ion/water homeostasis.

Across the diverse stress types discussed above, several common mechanistic themes emerge. HRW consistently enhances the activities of enzymatic antioxidants (SOD, POD, CAT, APX) and promotes the accumulation of non-enzymatic antioxidants (AsA, GSH), irrespective of the specific stress encountered (Yu et al., 2023; Wang et al., 2023; Song et al., 2023). Regulation of ion homeostasis, particularly through *SOS1*-mediated Na^+^ exclusion and vacuolar Na^+^/H^+^ exchanger 1(*NHX1)*-dependent vacuolar sequestration, represents a recurring mechanism in both salt and heavy metal stress responses (Wu et al., 2020, 2021a). ABA signaling is frequently activated under drought and temperature stress (Huang et al., 2026; Yan et al., 2022; Wang et al., 2023), whereas ethylene suppression constitutes a shared feature in postharvest preservation (Hu et al., 2014; Fang et al., 2024). These convergent mechanisms suggest that HRW confers broad-spectrum stress tolerance through a core set of conserved pathways (Li et al., 2022), which are further elaborated in Section 3.

## 3 Antioxidant and signal transduction mechanisms of hydrogen-rich water

**Figure 2:**
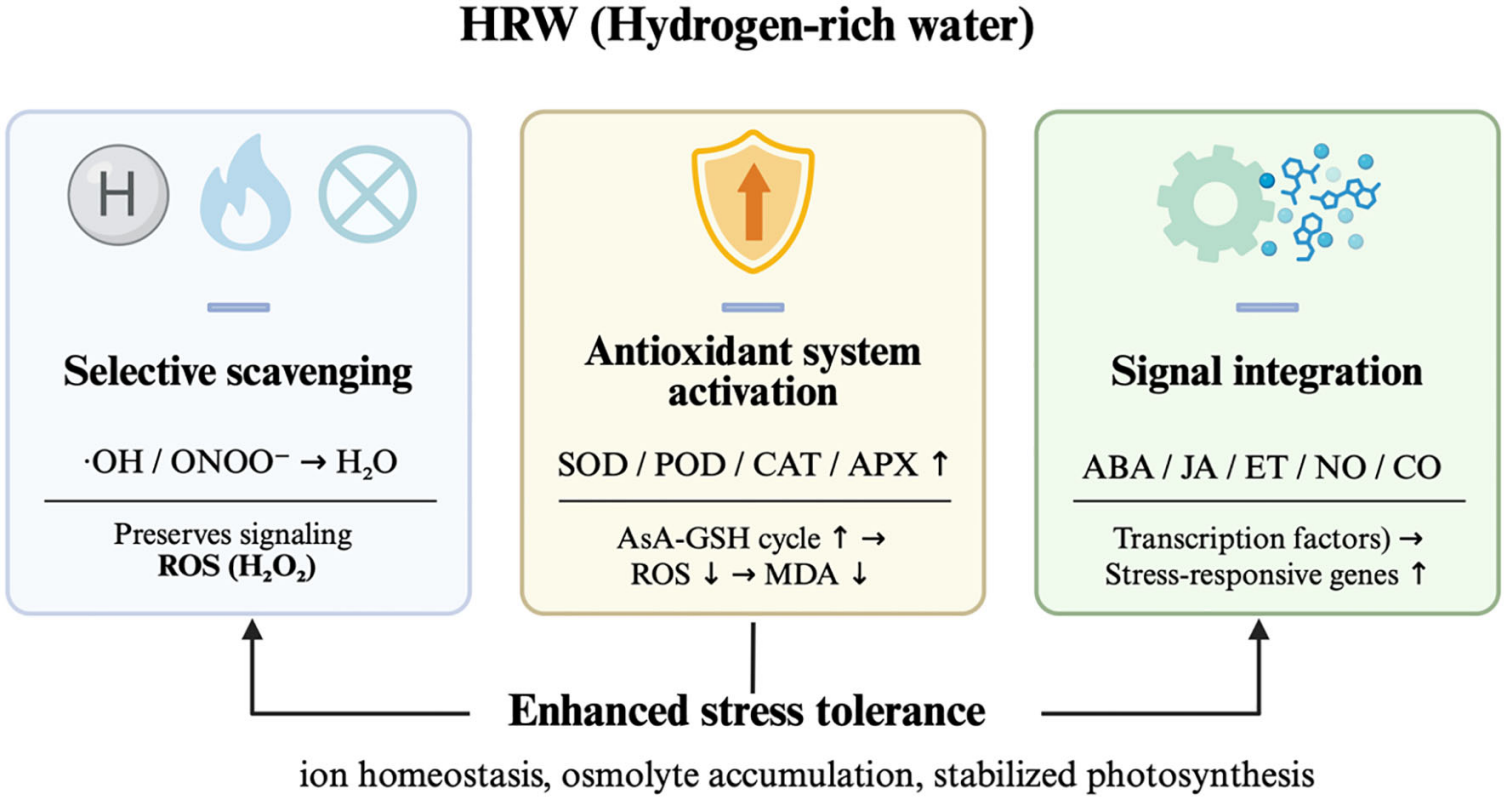
Schematic model of HRW-mediated stress tolerance mechanisms in plants. HRW (hydrogen-rich water) alleviates abiotic stress-induced oxidative damage through three major pathways: (1) direct selective scavenging of highly cytotoxic radicals (·OH and ONOO^−^) while preserving signaling ROS such as H_2_O_2_; (2) activation of the enzymatic antioxidant system (SOD, POD, CAT, APX) and the AsA-GSH cycle, leading to reduced ROS accumulation and membrane lipid peroxidation (MDA); and (3) regulation of signal transduction networks involving phytohormones (ABA, JA, ET) and gaseous signaling molecules (NO, CO), which further activate downstream transcription factors (*DREB*, *ZAT*10/*ZAT* 12) and stress-responsive gene expression. These three pathways coordinately maintain ion and redox homeostasis, protect chloroplast and mitochondrial function, and ultimately enhance plant stress tolerance.

The core biological property of molecular H_2_ lies in its selective antioxidant capacity. Ohsawa et al. (2007) first discovered that H_2_ selectively scavenges the most cytotoxic hydroxyl radical (·OH) and peroxynitrite (ONOO^−^) within cells, while having no significant effect on reactive oxygen species that play roles in signal transduction, such as H_2_O_2_ and superoxide anion (O_2_^−^). This property enables H_2_ to alleviate oxidative damage while preserving the physiological functions of ROS as signaling molecules, avoiding the potential side effects of traditional antioxidants that may interfere with normal signaling pathways (**Figure 1**). In plants, exogenous HRW treatment similarly acts through this mechanism, selectively scavenging excessive cytotoxic radicals under abiotic stresses such as salinity, heavy metal toxicity, and drought, thereby alleviating cellular oxidative damage (Li et al., 2022). A schematic model summarizing the major mechanisms by which HRW enhances plant stress tolerance is presented in **Figure 2**.

In addition to directly scavenging cytotoxic radicals, HRW treatment significantly activates the endogenous antioxidant defense system in plants. In terms of enzymatic antioxidant defense, HRW upregulates the activities and gene expression of SOD, POD, CAT, and APX, thereby enhancing the plant’s capacity to scavenge ROS (Yu et al., 2023; Wang et al., 2023). In terms of non-enzymatic antioxidant defense, HRW promotes the accumulation of ascorbate (AsA) and glutathione (GSH), strengthens the ascorbate-glutathione (AsA-GSH) cycle, and maintains cellular redox homeostasis (Song et al., 2023). Through the dual actions of direct scavenging and indirect activation, HRW effectively reduces MDA content, alleviates membrane lipid peroxidation, and protects the integrity of cell membrane structure and function.

**Figure 3:**
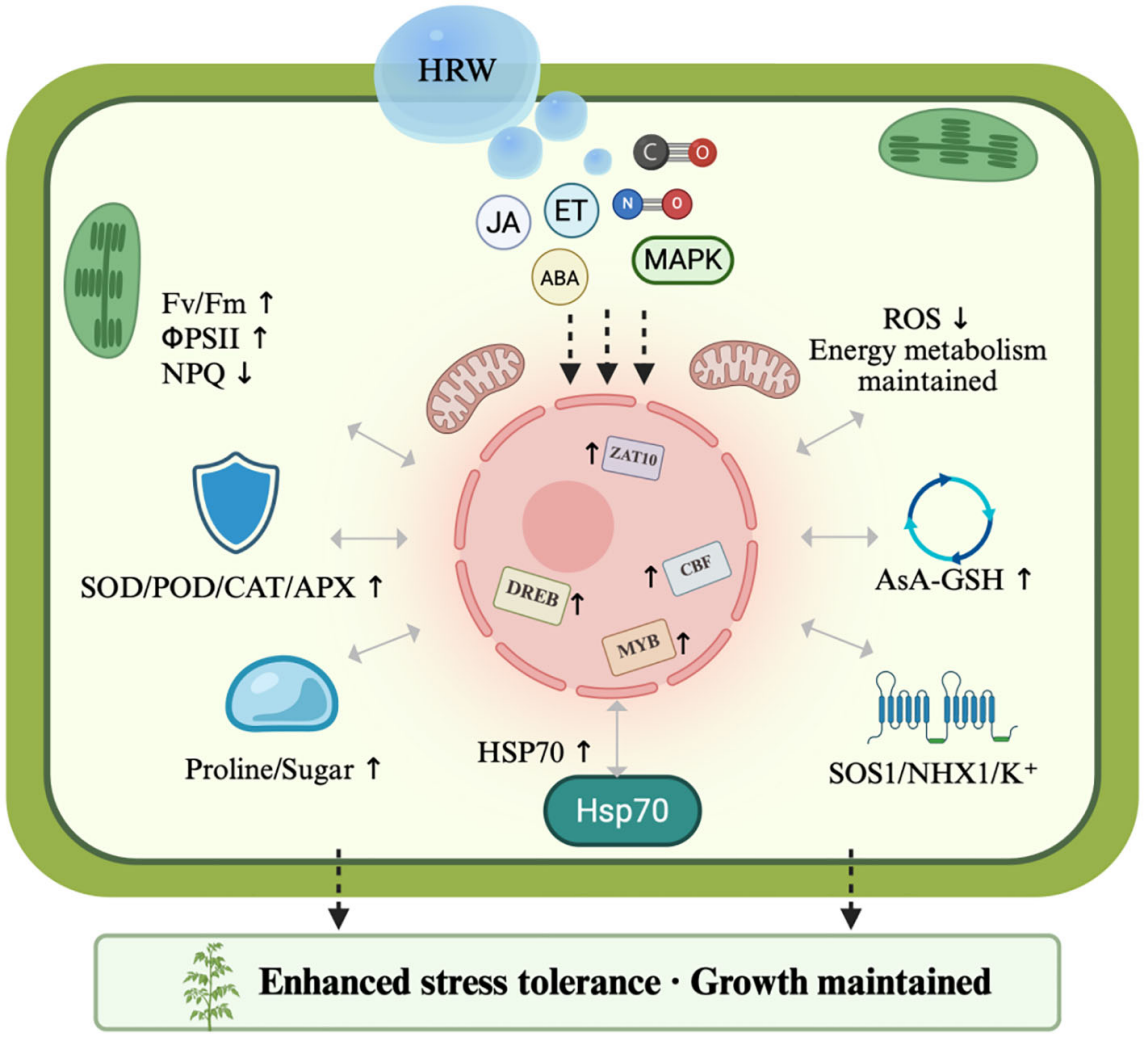
A panoramic view of HRW-mediated signaling and regulatory network within a plant cell. HRW enters the plant cell and activates a hierarchical regulatory network. In the cytoplasm, HRW triggers crosstalk with signaling molecules (NO, CO, ABA, JA, ET) and the MAPK cascade, which converge upon the nucleus. Within the nucleus, transcription factors (*ZAT*10/*ZAT* 12, *DREB*, *CBF*, *MYB*) are upregulated. These factors drive the expression of downstream effectors distributed throughout the cytoplasm, including antioxidant enzymes (SOD, POD, CAT, APX), the AsA-GSH cycle, osmolyte synthesis (proline, soluble sugars), ion transporters (*SOS1*, *NHX1*, K^+^ channels), and heat shock proteins (HSP70). Concomitantly, HRW protects chloroplasts (enhanced Fv/Fm and ΦPSII, reduced NPQ) and mitochondria (reduced ROS, maintained energy metabolism). The integrated outcome is enhanced stress tolerance and sustained growth.

HRW not only functions as an antioxidant but also acts as a gaseous signaling molecule involved in plant stress signal transduction. Studies have shown that H_2_ exhibits extensive crosstalk with phytohormone signaling pathways, including ABA, jasmonic acid (JA), and ethylene (ET). Under drought stress, HRW increases endogenous ABA content by regulating the expression of key ABA biosynthesis genes (*NCED*, *ZEP*, *AAO*), thereby inducing stomatal closure to reduce water loss (Huang et al., 2026; Yan et al., 2022). Under low-temperature stress, HRW activates the mitogen-activated protein kinase (MAPK) cascade and coordinates with ABA signaling pathways to regulate the expression of cold-responsive genes (Liu et al., 2022). Furthermore, H_2_ engages in signal crosstalk with other gaseous signaling molecules such as NO and CO to collectively regulate plant stress adaptation processes (Wu et al., 2021b; Zhao et al., 2021a). Transcriptomic analyses further reveal that HRW broadly alters the expression profiles of stress-responsive genes in plants, including those encoding antioxidant enzymes, osmolyte synthesis enzymes, ion transporters, and heat shock proteins. The upregulation of key transcription factors such as zinc finger transcription factor 10/12 (*ZAT10/ZAT12*) and dehydration-responsive element-binding protein (*DREB*) synergistically regulates the activation of downstream defense genes (Yu et al., 2023). Transcription factors serve as central hubs that integrate responses to diverse environmental stresses, with families such as NAM/ATAF/CUC transcription factor (*NAC*), *MYB*, *WRKY*, and ethylene response factor (*ERF*)/*DREB* playing crucial roles in coordinating stress signaling networks (Liu et al., 2024). Meanwhile, HRW exerts significant protective effects on key organelles such as chloroplasts and mitochondria, maintaining chloroplast structural integrity, protecting the photosynthetic electron transport chain, reducing mitochondrial ROS production, and sustaining cellular energy metabolism, thereby providing energy support for plant stress adaptation (Liu et al., 2022; Chen et al., 2017b) (**Figure 3**).

## 4 Conclusions and perspectives

### 4.1 Conclusions

Over the past decade, research on HRW in plant stress tolerance has grown rapidly, and a substantial body of evidence has now established a coherent mechanistic framework. HRW protects plants through a multi-layered mechanism that includes direct scavenging of ROS, activation of enzymatic and non-enzymatic antioxidant systems, and modulation of phytohormone and gasotransmitter signaling networks. These pathways converge to maintain ion and redox homeostasis, preserve chloroplast and mitochondrial function, and sustain plant growth under adverse environmental conditions.

A defining feature of HRW, and one that distinguishes it from conventional stress-relief agents, is its selective antioxidant capacity. It effectively neutralizes highly cytotoxic radicals such as ·OH and ONOO^−^ while leaving signaling ROS (e.g., H_2_O_2_, O_2_^−^) intact, thereby avoiding the nonspecific suppression of physiologically important ROS that is often associated with broad-spectrum antioxidants. This property, combined with its safety profile, low cost, and residue-free characteristics, positions HRW as a promising and sustainable strategy for agricultural stress management.

### 4.2 Future perspectives

Despite the progress summarized in this review, several challenges remain. A fundamental question is how plants perceive H_2_—no specific receptor has been identified to date, and chemical genetics screening combined with clustered regularly interspaced short palindromic repeats-CRISPR associated protein 9 (CRISPR-Cas9) validation may help uncover the sensing mechanism. At the systems level, HRW-triggered regulatory networks remain largely unexplored; integration of multi-omics datasets could reveal hub genes and regulatory nodes central to HRW-mediated stress tolerance. The crosstalk between H_2_ and other signaling molecules such as NO, H_2_S, and Ca²^+^ also requires further elucidation through targeted approaches using mutants defective in these pathways.

On the practical front, systematic dose–response studies are needed to establish evidence-based guidelines for HRW application across different crop species and stress types. Parallel field trials should evaluate whether HRW delivers consistent benefits under real-world conditions and whether it is economically viable for large-scale use. While improved delivery systems such as magnesium-based materials and nanobubble technology have shown promise in enhancing H_2_ stability, their cost-effectiveness and scalability remain to be demonstrated. Addressing these challenges will be essential to translate hydrogen agriculture into a practical tool for sustainable crop production.
